# Role of Si and C Impurities in Yellow and Blue Luminescence of Unintentionally and Si-Doped GaN

**DOI:** 10.3390/nano8121026

**Published:** 2018-12-10

**Authors:** Feng Liang, Degang Zhao, Desheng Jiang, Zongshun Liu, Jianjun Zhu, Ping Chen, Jing Yang, Shuangtao Liu, Yao Xing, Liqun Zhang

**Affiliations:** 1 State Key Laboratory of Integrated Optoelectronics, Institute of Semiconductors, Chinese Academy of Sciences, Beijing 100083, China; liangfeng13@semi.ac.cn (F.L.); dsjiang@semi.ac.cn (D.J.); zsliu@semi.ac.cn (Z.L.); jjzhu@semi.ac.cn (J.Z.); pchen@semi.ac.cn (P.C.); yangjing333@semi.ac.cn (J.Y.); lst7713@semi.ac.cn (S.L.); xingyao@semi.ac.cn (Y.X.); 2 Center of Materials Science and Optoelectronics Engineering, University of Chinese Academy of Sciences, Beijing 100049, China; 3 Suzhou Institute of Nano-tech and Nano-bionics, Chinese Academy of Sciences, Suzhou 215123, China; lqzhang2012@sinano.ac.cn

**Keywords:** GaN, yellow luminescence, blue luminescence, carbon impurity, Si impurity

## Abstract

Both yellow luminescence (YL) and blue luminescence (BL) bands of GaN films have been investigated for decades, but few works report the relationship between them. In this study, two sets of GaN samples grown via metalorganic chemical vapor deposition (MOCVD) were investigated. A close relationship was found between the YL and BL bands for unintentionally doped GaN and Si-doped GaN samples, both of which were grown without intentional acceptor doping. It was found that the intensity ratio of blue luminescence to yellow luminescence (*I_BL_*/*I_YL_*) decreases sharply with the increase in carbon impurity concentration, even though both *I_BL_* and *I_YL_* increase obviously. It was also found that *I_BL_*/*I_YL_* decreases sharply with the increase in Si doping concentration. It is suggested that the C and Si impurities play important role in linkage and competition of the blue and yellow luminescence.

## 1. Introduction

III-nitrides, such as GaN, InGaN, AlGaN and InAlGaN, are the key materials of third-generation semiconductors. Their photonic and electronic devices, such as light-emitting diodes (LEDs) [1,2], laser diodes (LDs) [3,4,5], photodetectors (PDs) [6,7] and high electron mobility transistors (HEMTs) [8,9], have extensive applications in solid state lighting, light or microwave communication, and power electronics. Investigations on the GaN-based materials and devices have obtained great achievements in recent decades. However, some problems related to its basic material properties are still not completely solved. For example, it is widely known that the yellow luminescence (YL) band peaking around 2.2 eV and the blue luminescence (BL) band peaking around 2.9 eV from the GaN films are mainly caused by the carrier recombination between the donor–acceptor pairs (DAPs). However, the mechanism of the YL band is quite complicated [10,11,12,13,14,15], and the origin of the blue luminescence (BL) band peaking around 2.9 eV in GaN grown without intentional acceptor doping is also in dispute [16,17,18,19,20,21]. Which impurity and/or native point defect introduces the donor–acceptor pair luminescence has not been confirmed affirmatively. Moreover, only relatively few research works have reported about the relationship between the BL and YL bands in GaN materials [22]. Moreover, our previous works reported the influence of C impurity on the performance of GaN-based devices. For the GaN-based photodetectors, it was found that the residual C impurities in the i-layer has a strong influence on the spectral responsivity, and the performance of the photodetectors can be improved by reducing the residual C impurity concentration of the i-GaN layer [23,24]. For the GaN-based light emission diode (LED), it was also found that reduction of the residual C concentration in the InGaN/GaN multiple quantum well can enhance the peak intensity of electroluminescence by decreasing the C related non-radiative recombination centers in InGaN layers [25]. Therefore, our previous works on the influence of impurities on the devices are enough to demonstrate the meaning of this work. In this study, two sets of GaN films with steplike increasing C and Si impurity concentrations were investigated systematically by photoluminescence (PL) and secondary ion mass spectroscopy (SIMS), and the relationship between the BL and YL in GaN was elucidated. It was found that the integrated intensities of the two bands may be closely related to each other, and the C and Si impurities play an important role in linking the BL and YL band of GaN films.

## 2. Materials and Methods

**Figure 1:**
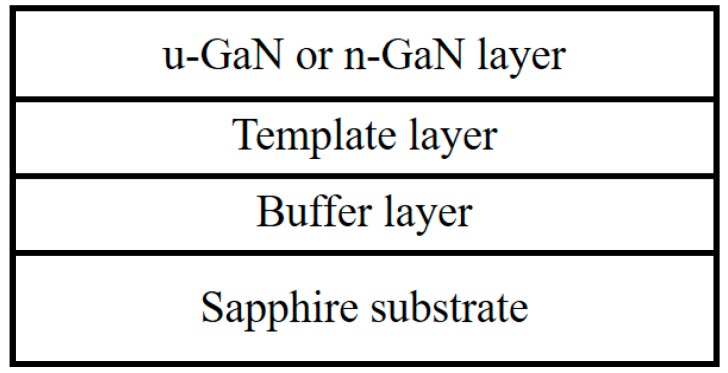
Schematic diagram of the epitaxial structure of GaN samples.

The GaN samples were grown on c-plane sapphire substrate by metal organic chemical vapor deposition (MOCVD) equipment of Aixtron, Germany. Ammonia (NH_3_) and trimethylgallium (TMGa) were the precursors of N and Ga, respectively, and SiH_4_ was used as the Si dopant. The schematic diagram of the epitaxial structure of GaN samples is shown in Figure 1, and a two-step growth process was used to grow them. First, a low-temperature GaN layer (LT GaN) and a high-temperature GaN template layer (HT GaN) was deposited on the sapphire substrate successively. Then, an unintentionally doped GaN layer (u-GaN) or Si doped GaN layer (n-GaN) was grown at high temperature. The thicknesses of LT GaN, HT GaN and the top u-GaN or n-GaN film were around 20 nm, 1 μm and 1 μm, respectively. The thicknesses of the layers (LT-GaN, HT-GaN and the top u-GaN or n-GaN) were determined according to the in situ optical reflectance curves measured during the MOCVD growth. By this method, the growth rate can be obtained first, and then the thickness can be calculated using the growth rate and growth time. The growth conditions were the same for LT GaN and HT GaN, but different for the top GaN films. Two sets of GaN samples were prepared in this study, and their growth conditions are listed in Table 1. Series I included nine u-GaN film samples, A0–I0. The SiH_4_ flow rate for the top GaN layer growth was always kept zero, but other growth conditions, such as temperature, pressure or NH_3_ flow rate, were intentionally modulated to change the residual C doping concentration in these layers. The detailed MOCVD growth conditions can be found in our previous report [26]. For Series II 4 n-GaN samples A0, A1, A2 and A3, the top GaN layers were grown with intentional Si doping by using different SiH_4_ flow rates (0, 0.03, 0.3 and 1.6 L/min) and other growth parameters were the same as used for sample A0. In Table 1, [C] is residual carbon C concentration and [Si] is Si doping concentration.

**Figure 2:**
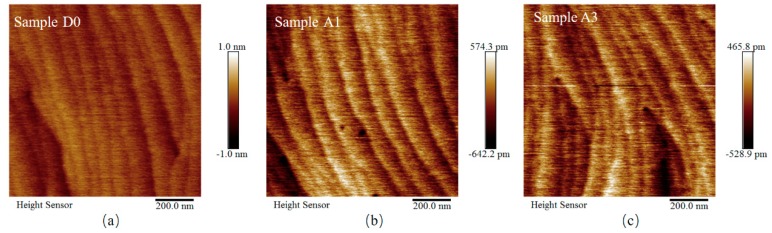
Atomic force microscopy surface images of samples D0, A1 and A3 in 1 μm × 1 μm regions. Color bar is shown at the right side of each figure.

In addition, atomic force microscopy (AFM) measurements (Bruker, Germany) for samples D0, A1 and A3 were taken to check the morphology of our u-GaN and n-GaN samples in a way of selective examination. In Figure 2, in the 1 μm × 1 μm regions of samples D0, A1 and A3, the feature of step flow can be seen clearly in the surface topography, and the root-mean-square (RMS) roughnesses of samples D0, A1 and A3 were 0.13, 0.18 and 0.14 nm, respectively. These AFM images clearly indicate that our GaN samples have excellent surface morphology, even where the total epilayer thickness is as high as around 2 μm.

Room temperature (RT) and temperature dependent photoluminescence (PL) measurements of the GaN samples were carried out using the 325 nm line of a He-Cd laser at an excitation density of about 0.4 W/cm^2^, and the luminescence intensity was normalized by the intensity of the near-band-edge emission peak, i.e., UVL band.. Meanwhile, secondary ion mass spectroscopy (SIMS) (ATOMIKA 4500, Oberschleißheim, Germany), measured with Cs^+^ ions as the primary source, was taken to check depth profiles of hydrogen, C, oxygen and Si impurities concentrations ([H], [C], [O] and [Si], respectively). The raster size was 80 µm × 80 µm and the collected area (in diameter) was 30 µm, located around the centers of 2-inch n-GaN wafers. For the Hall measurement, the electrical resistivity of the GaN sample was measured using the van der Pauw method at 300 K. GaN samples were first cut into square with size 1 cm × 1 cm, and then the metal indium was used to form ohmic contact with GaN samples. During Hall measurement, the employed current and magnetic field intensity were kept at 0.2 mA and 5000 Gs, respectively.

## 3. Results and Discussion

**Figure 3:**
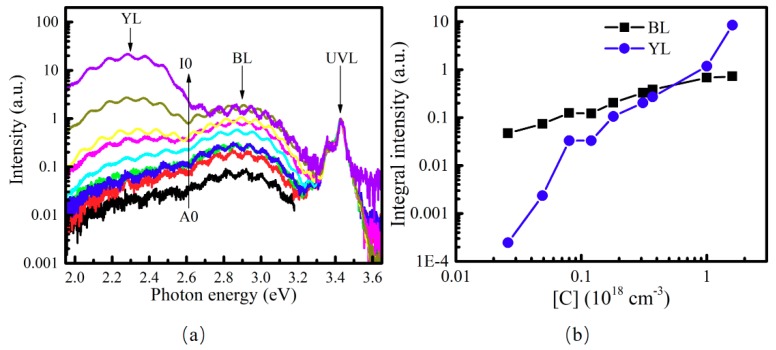
(**a**) Normalized room temperature PL spectra; and (**b**) the dependences of integral intensities of BL (square) and YL (circle) bands on the C concentration based on the data points of samples A0–I0 (from left to right).

First, the influence of C impurity on BL and YL bands were examined. Series I samples A0–I0 were prepared for this purpose. Their growth conditions weren intentionally modified to change the residual C concentration incorporated into the GaN films during the growth. The SIMS measurement results (the profile curves are not shown here) demonstrate that the C concentration in samples A0–I0 stepwise increased: 2.6 × 10^16^ cm^−3^, 4.9 × 10^16^ cm^−3^, 8.6 × 10^16^ cm^−3^, 1.2 × 10^17^ cm^−3^, 1.8 × 10^17^ cm^−3^, 3.1 × 10^17^ cm^−3^, 3.7 × 10^17^ cm^−3^, 1.0 × 10^18^ cm^−3^ and 1.6 × 10^18^ cm^−3^, respectively. Meanwhile, the oxygen impurity concentration in samples A0–I0 was found to be at a similar level, i.e., 6.0 × 10^16^ cm^−3^, and the hydrogen impurity concentration varied slightly from 2.6 × 10^16^ cm^−3^ to 4.8 × 10^17^ cm^−3^. Figure 3a shows the room temperature PL spectra of samples A0–I0 where the intensity scale was normalized according to the intensity of near-band-edge ultraviolet emission (UVL) peak. There were three distinct emission bands in RT PL spectra of samples A0–I0, i.e., near-band-edge ultraviolet emission (UVL) peak, blue luminescence (BL) band and yellow luminescence (YL) band. Their peak energies were located around 3.4, 2.9 and 2.3 eV, respectively. It was found that both peak intensities of the BL and YL bands increased with the increase of the C impurity. In addition, it was found that the intensity of BL peak was larger than that of UVL when the C impurity concentration was ≥3.7 × 10^17^ cm^−3^ (sample G0), and the YL intensity peak became larger than those of BL and UVL when the C impurity concentration was 1.6 × 10^18^ cm^−3^ (sample I0). In general, the obtained result indicates that an increase of C impurity concentration in samples A0–I0 can enhance both BL and YL band.

Moreover, the dependences of BL and YL band integral intensities on C concentration were quite different for samples A0–I0, as shown by the dependences of integral intensities of BL (*I_BL_*) and YL (*I_YL_*) bands on the C concentration in Figure 3b.Thesecondary ion mass spectroscopy results of carbon impurity in samples A0–I0 can be found in Figure S1.It can be seen that *I_BL_* value increased from 0.047 to 0.72, and the *I_YL_* value increased much more quickly, from 2.46 × 10^−4^ to 8.51, when the C concentration increased from 4.9 × 10^16^ cm^−3^ to 1.6 × 10^18^ cm^−3^. In addition, it was found that *I_BL_* value was larger than *I_YL_* value when the C concentration was less than 1.6 × 10^18^ cm^−3^, but became smaller than *I_YL_* after the C concentration increased further (sample H0 and I0).

**Figure 4:**
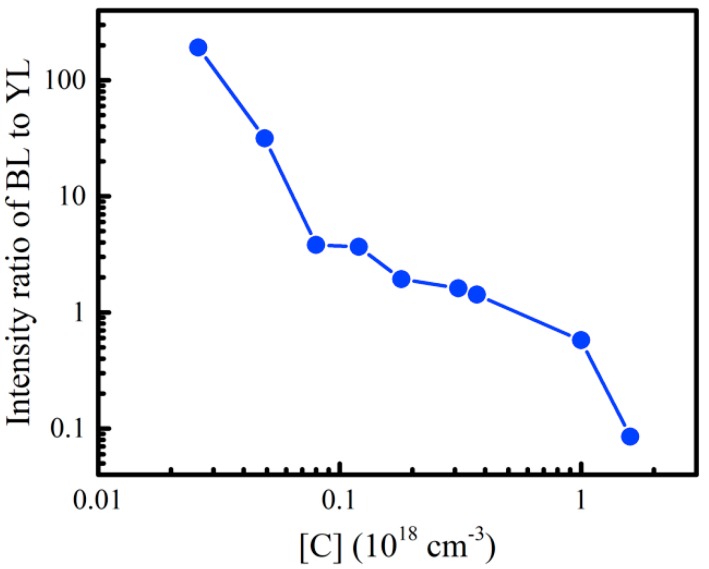
Dependences of the intensity ratio of BL to YL (circles) of u-GaN samples A0–I0 on the C concentration. The blue line in Figure 4 is used to guide the eye.

Meanwhile, Figure 4 shows the dependence of the ratio of BL to YL integral intensities (*I_BL_*/*I_YL_*) on C concentration. The *I_BL_*/*I_YL_* value of sample A0 decreased sharply from about 191.21 to 0.085 when the C concentration increased from 4.9 × 10^16^ cm^−3^ to 1.6 × 10^18^ cm^−3^. It means that C impurity has an influence on the ratio of BL and YL bands in u-GaN samples A0–I0. It is known that C is one of the most important impurity-induced defects in in MOCVD-grown GaN and other nitride materials, which is unintentionally introduced during the epitaxial growth with MOCVD, mainly due to the presence of C in the employed organic gas precursors [27,28,29]. In addition, it is widely known that the C impurity in GaN acts not only as a shallower acceptor, but may also be a deeper acceptor. It has been proposed that the C related defects, such as the C atom substituting for a nitrogen site (C_N_), the complex defect of C_N_ and O_N_ (C_N_-O_N_) or the complex of V_Ga_ and C_N_ (V_Ga_-C_N_), may all be responsible for the acceptors in the DAP luminescence of BL [15,16] and YL [17,30,31,32] bands in GaN. In a recent work, Reshchikov et al. proposed that both the BL1 and YLc bands originate from the acceptor states of C _N_, and they are related with the electron transitions via the −/0 and 0/+ thermodynamic transition levels of the C_N_, respectively [22]. According to the theoretical calculation and experimental results, the C related acceptor levels for the BL and YL bands are located around 0.93 eV (*E_A_*_1_) and 0.36 eV (*E_A_*_2_) above the valence band, respectively, where *E_A_*_1_ and *E_A_*_2_ are deeper and shallower acceptor levels. Thus, a C-concentration related change of *I_BL_*/*I_YL_* is understandable if it is assumed that the observed BL and YL luminescence bands come from donor–acceptor pair (DAP) transitions related to C induced acceptor levels *E_A_*_1_ and *E_A_*_2_. We think this model is reasonable and is well supported by our experimental results.

For samples A0–I0, the C related acceptor level (*E_A_*_1_) may capture electrons from the native defect-related donor level (*E_D_*_1_), forming the YL channel luminescence. The second C related acceptor level (*E_A_*_2_) may capture electrons from the native defect-related donor level (*E_D_*_2_), resulting in the BL channel luminescence. It is interesting to note that, when C concentration increased, both *I_BL_* and *I_YL_* values increased, but *I_BL_*/*I_YL_* decreased significantly, suggesting the C concentration may influence relative concentrations or charging states of *E_A_*_1_ and *E_A_*_2_ by changing the position of Fermi level of GaN samples. This would play an important role in influencing competitive process between the BL and YL transition channels. The YL seems to be a preferable recombination channel compared to the BL one when the C concentration is high enough. On the one side, the above discussion shows that two different C-related acceptor levels *E_A_*_1_ and *E_A_*_2_ of C_N_ may be responsible for the YL and BL bands, respectively. The position of Fermi level may greatly influence the charging states of *E_A_*_1_ and *E_A_*_2_, and thus the relative intensity of YL and BL bands. On the other side, it is reported that other C-related complex may also contribute to YL and BL luminescence bands [16,31,32,33]. According to theoretical calculations by the first-principles, the formation energies of the C related defect levels sensitively depend on the Femi energy level (*E_F_*) [34,35,36]. Thus, Hall measurements were taken to check the position of the Femi energy level and the electrical resistivity. It was found that, for example, the resistivities of samples A0, C0 and D0 were 3.2 Ω·cm, 2.4 × 10^3^ Ω·cm, 4.0 × 10^3^ Ω·cm, respectively. The resistivities of other samples with higher C concentrations were even semi-insulating. It suggests a trend that the electron concentration decreases and the Femi energy level moves farther away from the conduction band when the C impurity concentration increases. It indicates that the charging states of these two C related acceptor levels *E_A_*_1_ and *E_A_*_2_ will be changed (or the relative concentrations of *E_A_*_1_ and *E_A_*_2_ may be changed) when the C impurity concentration varies. Figure 3 and Figure 4 show that *I_BL_*/*I_YL_* value decreased significantly with increasing C concentration, although both *I_BL_* and *I_YL_* were enhanced at the same time. Therefore, it is proposed that more electron transits via the −/0 thermodynamic transition levels *E_A_*_1_ of the C_N_ (or more shallower acceptor level defects are formed than deeper ones), enhancing the YL band when the C concentration becomes high enough. Finally, the YL channel was much more competitive and enhanced more quickly than BL, and the *I_BL_*/*I_YL_* value decreased significantly when the C concentration increased from 2.6 × 10^16^ cm^−3^ to 1.6 × 10^18^ cm^−3^.

**Figure 5:**
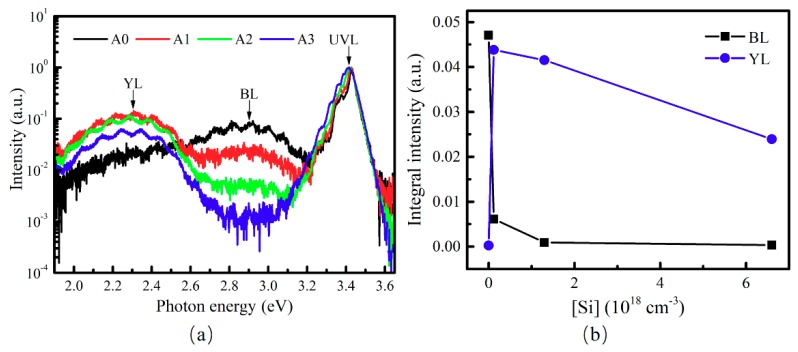
(**a**) Normalized room temperature PL spectra; and (**b**) the dependences of integral intensities of BL (square) and YL (circle) bands on the Si concentration based on the data points of samples A0, A1, A2 and A3 (from left to right).

To study the relationship between the BL and YL further, GaN samples A0–A3 in Series II were prepared. These four samples had similar concentration levels of residual hydrogen, C and oxygen impurities, but the doping level of intentionally doped Si impurity varied. During MOCVD growth, the SiH_4_ flow rate varied for samples A0–A3, being 0, 0.03, 0.3 and 1.6 L/min, respectively. The SIMS results (the profile curves are not shown here) confirm that the concentrations of hydrogen, C and oxygen impurities are almost the same in these samples: 2.8 × 10^17^ cm^−3^, 2.6 × 10^16^ cm^−3^ and 6.0 × 10^16^ cm^−3^, respectively. On the other hand, the Si concentrations in samples A0–A3 were 0, 1.2 × 10^17^ cm^−3^, 1.3 × 10^18^ cm^−3^ and 6.6 × 10^18^ cm^−3^, respectively. In Figure 5a, the room temperature PL spectra of samples A0–A3 show that the peak intensities of the BL and YL bands were lower than the UVL intensities of samples A0–A3. In addition, it was found that the peak intensity of BL band decreased significantly when the SiH_4_ flow rate increased from 0 to 1.6 L/min. It almost vanished when the SiH_4_ flow rate reached 1.6 L/min. Meanwhile, the YL intensity was enhanced after Si doping, and remained much larger than the BL intensity. Furthermore, as shown in Figure 5b, which depicts the dependences of integral intensities of BL (*I_BL_*) and YL (*I_YL_*) bands on the Si concentration of samples A0–A3, the *I_BL_* values were 0.047, 6.12 × 10^−3^, 9.27 × 10^−4^ and 3.41 × 10^−4^, respectively, and the *I_YL_* values where 2.46 × 10^−4^, 0.044, 0.042 and 0.024, respectively. It indicates that *I_BL_* value decreased by at least one order of magnitude after Si doping, and the *I_YL_* value increased obviously by almost two orders of magnitude after the doping of Si. It demonstrates that doping Si in GaN can greatly weaken the BL, and enhance the YL.

**Figure 6:**
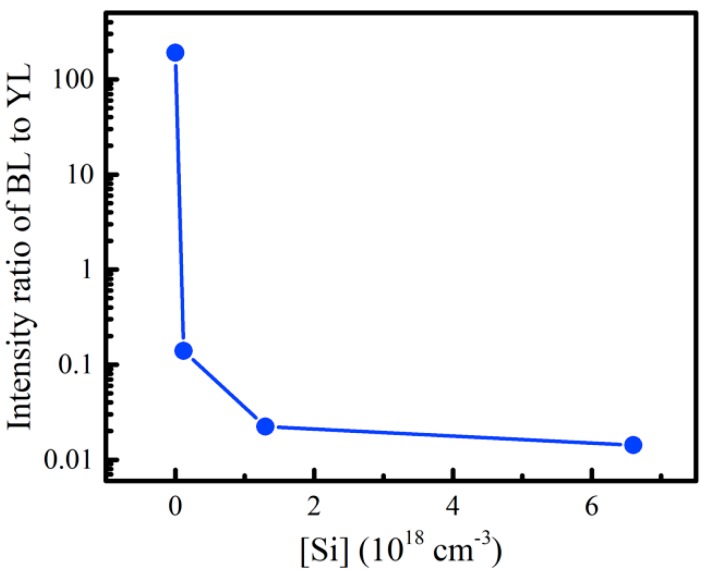
Dependences of the intensity ratio of BL to YL of n-GaN samples A0–A3 on the Si concentration. The blue line is used to guide the eye.

Figure 6 shows the dependence of the integral intensity ratio of BL to YL (*I_BL_*/*I_YL_*) of samples A0–A3 on the Si concentration, and the secondary ion mass spectroscopy results of Si concentration in samples A1–A3 can be found in Figure S2. The *I_BL_*/*I_YL_* values for samples A0–A3 were 191.21, 0.14, 0.022 and 0.014, respectively. It is obvious that the *I_BL_*/*I_YL_* value decreased sharply with the increase of Si doping concentration. It is widely known that Si dopant in GaN acts as a shallow donor and the Si doping can introduce a large amount of Si donor level. The experimental result indicates that the Si donor level is largely involved in donor–acceptor pair recombination of YL. It is implied that, when the same C impurity is introduced during the GaN growth, the photogenerated electrons in Si-doped samples may prefer to relax to the Si donor level, and enhance the YL. Such an electron relaxation process may result in a suppression of the BL, suggesting that only a small part of photo-generated electrons will be able to enter the BL transition channel. It means that the DAP transition of BL channel may be not related with Si donors. On the contrary, the increase of Si donor level may suppress BL, playing an important role in influencing competitive relation between the BL and YL.

**Figure 7:**
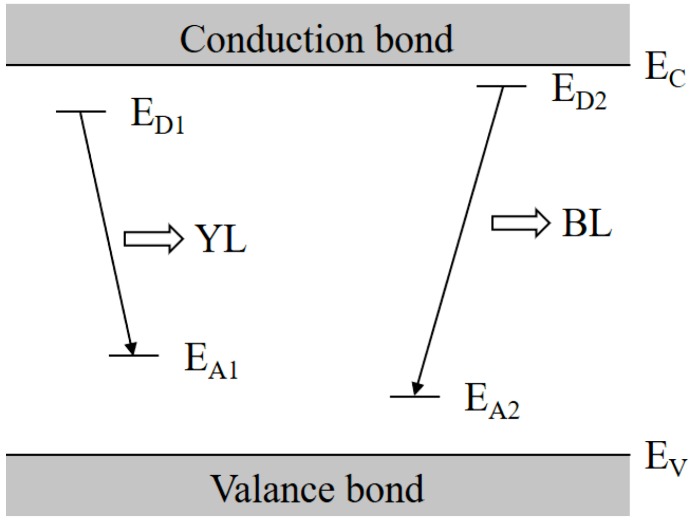
Schematic diagram of the proposed transition model on the YL and BL of u-GaN samples. where *E_D_*_1_ is mainly contributed by Si donor.

Based on the above results, a transition model about the YL and BL bands of our GaN samples is proposed and is schematically shown in Figure 7, in which both YL and BL are DAP luminescence processes, but they are related to the different donor and acceptor pairs, i.e., *E_D_*_1_ and *E_A_*_1_, and *E_D_*_2_ and *E_A_*_2_, respectively. Both *E_A_*_1_ and *E_A_*_2_ acceptor levels are induced by C impurity, but their depth is different, and the acceptor level *E_A_*_2_ is assumed to be much shallower than *E_A_*_1_. On the other hand, the donor level of *E_D_*_2_ is also shallower than *E_D_*_1_, and *E_D_*_1_ is mainly contributed by Si donors. In detail, for the unintentionally C-doped GaN, i.e., samples A0–I0, the YL and BL could be enhanced by the C related acceptors *E_A_*_1_ or *E_A_*_2_. Meanwhile, more electron transits via the −/0 thermodynamic transition levels of the C_N_ when the C concentration becomes high enough. Consequently, the *I_BL_*/*I_YL_* value decreases with the increase in C concentration. On the other hand, when the same C impurity is introduced during the GaN growth, with the increase in Si density more DAPs between the *E_A_*_1_ and *E_D_*_1_ are formed and *I_YL_* is enhanced, thus both *I_BL_* and *I_BL_*/*I_YL_* are reduced. Therefore, the C and Si impurities are important for linking the intensities of BL and YL in u-GaN and n-GaN materials.

Moreover, it is interesting to note that the effect of Si doping on the photoluminescence is not only its contribution to the increase of *E_D_*_1_ level of Si donor, but also its influence on the shift of Fermi level and thus the integral intensity of YL. As shown in Figure 5b, the *I_YL_* values of samples A1–A3 decreased from 0.044 to 0.024 when the Si doping concentration increased from 2 × 10^17^ cm^−3^ to 6.6 × 10^18^ cm^−3^. On the other hand, Hall measurements of these n-GaN samples showed that the electrical resistivities of A1–A3 were 0.37 Ω·cm, 0.018 Ω·cm, 0.0047 Ω·cm, respectively. It means that the electron concentration of samples A1–A3 increases and the Femi energy level moves towards the conduction band with the increase of Si doping. It indicates that, in this case, the charging states of two C related C_N_ acceptor levels will be changed, and less *E_A_*_1_ levels are formed, thus the YL channel is weakened and integral YL intensity decreases when the Si doping concentration increases, and even more Si-related *E_D_*_1_ levels are formed. That is why, even though the *I_YL_* values of Si-doped GaN (samples A1–A3) are larger than that of undoped GaN (samples A0), the *I_YL_* value decreases when Si doping concentration increases for A1–A3.

## 4. Conclusions

In summary, the relationship between the C-related BL and YL bands in u-GaN and Si-doped films was investigated. It was found that the integrated intensities of these two bands may be closely related to each other. Both are related to C acceptors and increase with the increase of C doping concentration, but at different speeds. In addition, BL band is weakened, and the YL band is enhanced after intentional Si doping. This suggests that both C and Si impurities play important role in linkage and competition of the BL and YL.

## Figures and Tables

**Table 1:** Growth conditions of u-GaN and n-GaN samples, where [C] is residual C concentration, and [Si] is Si doping concentration.

Sample	SiH_4_ Flow Rate (L/min)	[Si] (cm^−3^)	[C] (cm^−3^)
A0	0	0	2.6 × 10^16^
B0	0	0	4.9 × 10^16^
C0	0	0	8.6 × 10^16^
D0	0	0	1.2 × 10^17^
E0	0	0	1.8 × 10^17^
F0	0	0	3.1 × 10^17^
G0	0	0	3.7 × 10^17^
H0	0	0	1.0 × 10^18^
I0	0	0	1.6 × 10^18^
A1	0.03	1.2 × 10^17^	2.6 × 10^16^
A2	0.3	1.3 × 10^18^	2.6 × 10^16^
A3	1.6	6.6 × 10^18^	2.6 × 10^16^

## Supplementary Materials

The following are available online at http://www.mdpi.com/2079-4991/8/12/1026/s1, Figure S1: Secondary ion mass spectroscopy results of carbon impurity in samples A0–I0, Figure S2: Secondary ion mass spectroscopy results of Si concentration in samples A1–A3.
