# Supplementary material for: Role of Si and C Impurities in Yellow and Blue Luminescence of Unintentionally and Si-Doped GaN
Source: Nanomaterials (Basel). 2018 Dec 10;8(12):1026. doi: 10.3390/nano8121026 (PMC6316107; doi:10.3390/nano8121026)
Supplement: Supplementary file 1 [file nanomaterials-08-01026-s001.pdf]

Article

Role of Si and C Impurities in Yellow and Blue Luminescence of Unintentionally and Si-Doped GaN

Feng Liang [1], Degang Zhao [1,2,*], Desheng Jiang [1], Zongshun Liu [1,2], Jianjun Zhu [1], Ping Chen [1], Jing Yang [1], Shuangtao Liu [1], Yao Xing [1] and Liqun Zhang [3]

[1] State Key Laboratory of Integrated Optoelectronics, Institute of Semiconductors, Chinese Academy of Sciences, Beijing 100083, China; liangfeng13@semi.ac.cn (F.L.); dsjiang@semi.ac.cn (D.J.); zsliu@semi.ac.cn (Z.L.); jjzhu@semi.ac.cn (J.Z.); pchen@semi.ac.cn (P.C.); yangjing333@semi.ac.cn (J.Y.); lst7713@semi.ac.cn (S.L.); xingyao@semi.ac.cn (Y.X.)

[2] Center of Materials Science and Optoelectronics Engineering, University of Chinese Academy of Sciences, Beijing 100049, China

[3] Suzhou Institute of Nano-tech and Nano-bionics, Chinese Academy of Sciences, Suzhou 215123, China; lqzhang2012@sinano.ac.cn

* Correspondence: dgzhao@red.semi.ac.cn; Tel.: +86-10-8230-4312

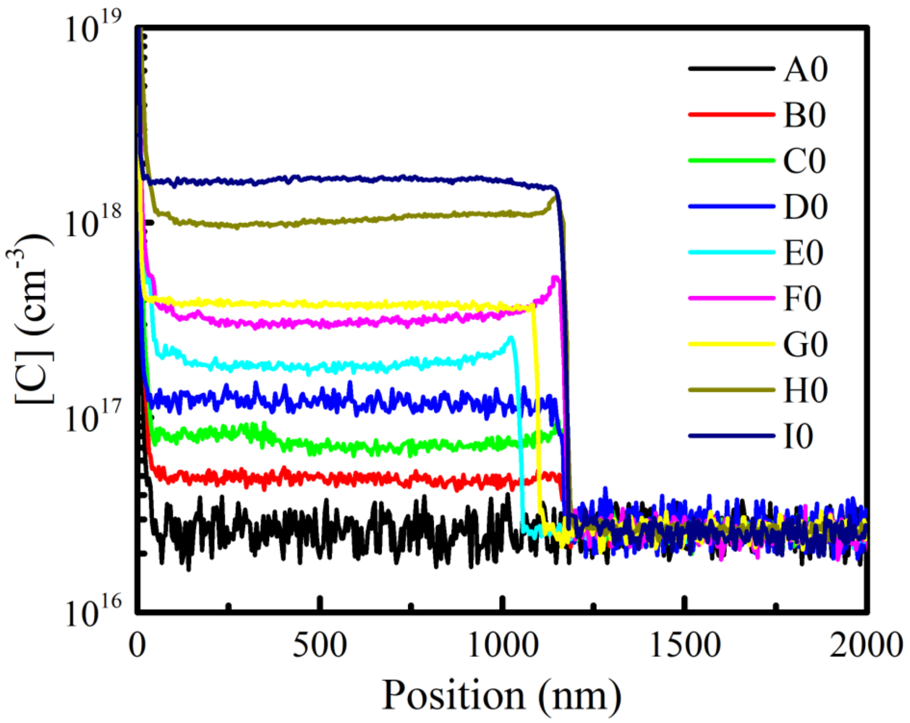

Figure S1. Secondary ion mass spectroscopy results of carbon impurity in samples A0–I0.

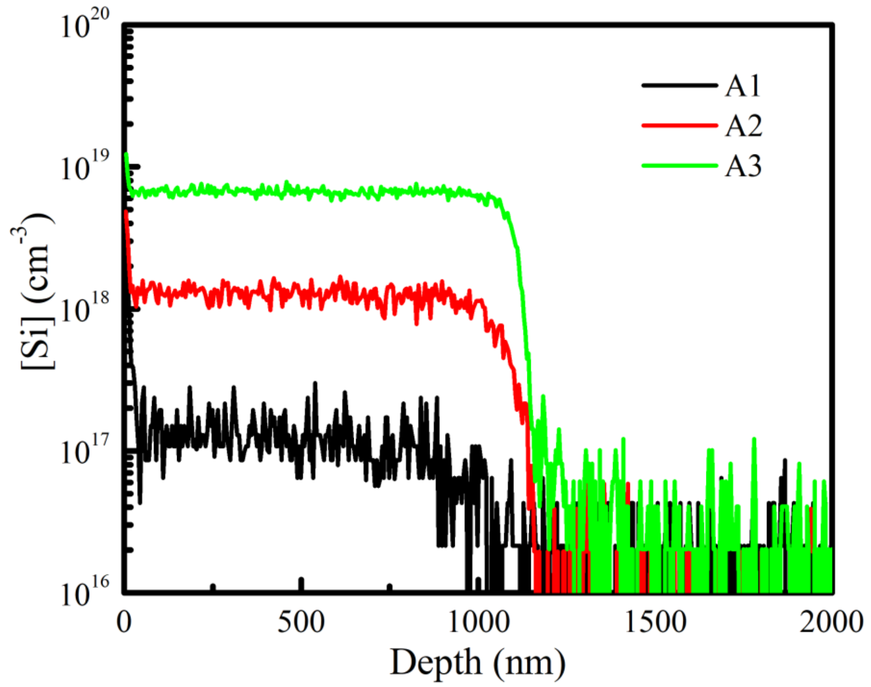

Figure S2. Secondary ion mass spectroscopy results of Si concentration in samples A1–A3.
